# When knowledge flows backward: enhancing older employee creativity through cognitive flexibility

**DOI:** 10.3389/fpsyg.2026.1863257

**Published:** 2026-07-22

**Authors:** Yangchun Yan, Panpan Ren

**Affiliations:** School of Business Administration, Xinjiang College of Science & Technology, Korla, China

**Keywords:** aging workforce, cognitive flexibility, knowledge counterflow, knowledge stickiness, older employee creativity

## Abstract

With the trend of aging workforce, management practitioners and scholars share a strong interest in understanding how to harness creative potential of older employees. In this study, we seek to address this issue by exploring the factors that enhance older employee creativity. We argue that knowledge counterflow, i.e., transfer of knowledge from younger employees to their older counterparts, helps facilitate older employee creativity. We propose that knowledge counterflow may increase older employees' cognitive flexibility and facilitate their creativity. This research further examines the moderating role of knowledge stickiness, suggesting that the positive effect of knowledge counterflow can be weaker when knowledge is less absorbable, i.e., knowledge stickiness is at higher level. A time-lagged survey study was conducted and data collected from a sample of 323 older employees. The findings provided support for our research model, contributing to the literature on human resource development for the aging workforce.

## Introduction

Against the backdrop of profound global demographic shifts, workforce aging has become an unavoidable strategic issue in the field of organizational management (Pepe et al., 2026). According to United Nations data, by 2050, the proportion of the global population aged 60 and above is projected to reach 22%. Against this trend, how to effectively activate the creative potential of older employees (typically defined as those aged 40 and above) has become both a key to maintaining organizational competitiveness and a practical challenge for human resource management (Kitz et al., 2025). Regarding the relationship between age and creativity, the existing literature presents a seemingly contradictory picture. On the one hand, a widely held stereotype suggests that older employees are rigid in their thinking and have difficulty generating novel ideas (Rothermund and Brandtstädter, 2003); on the other hand, empirical research indicates that creativity does not inevitably decline with age (Ng and Feldman, 2013). Instead, it evolves, drawing upon richer life experiences and deeper domain expertise, with older employees even demonstrating unique advantages in radical innovation (Murmann et al., 2023). This contradiction suggests that the creative potential of older employees objectively exists; the question lies in how to activate this potential through appropriate organizational support mechanisms.

This study proposes that knowledge counterflow—the process by which knowledge flows from younger to older employees—may be one of the key mechanisms for activating older employee creativity. Unlike traditional top-down knowledge transfer (from senior to novice), knowledge counterflow exposes older employees to emerging knowledge domains such as information technology and cutting-edge methods (Fasbender and Gerpott, 2022). The input of such non-redundant knowledge may precisely break the cognitive entrenchment that older employees develop through long-term reliance on existing experience (Dane, 2010; Li et al., 2021), thereby providing a cognitive opportunity for the emergence of their creativity. Furthermore, we point out that the effect of knowledge counterflow on enhancing creativity is not unconditional. When the knowledge conveyed by younger employees is difficult to understand and absorb (i.e., when knowledge stickiness is high), older employees may not be able to effectively utilize these knowledge resources (Ganguly et al., 2019). Therefore, this study integrates cognitive flexibility theory with the knowledge management literature to construct a moderated mediation model, aiming to answer: “when” and “how” knowledge counterflow facilitates older employee creativity.

In this study, we propose that a key factor for facilitating older employee creativity is knowledge counterflow, i.e., the process by which knowledge transfers from younger to older employees (Burmeister et al., 2020; Fasbender and Wang, 2017). This study draws on cognitive flexibility theory to argue that knowledge counterflow can enhance cognitive flexibility and thus increase older employee creativity. The theory suggests that, as people get to learn a new complex domain of knowledge, they are aware of the new frameworks of thinking (Martin and Rubin, 1995; Spiro, 1988). The information and knowledge held in different generations differs in terms of scope and structure (Fasbender and Gerpott, 2022). As such, knowledge transferred from younger employees may introduce new ways of thinking; the recent rapid development of new technologies makes it even more likely to occur (Ahmad et al., 2022; Beane and Anthony, 2024). Seeking to gain a deeper understanding, the older employees tend to adapt their thinking to the new structure and framework (Spiro, 1988). This enables them to take a new perspective, thus facilitating their creativity (Anderson et al., 2014). We therefore suggest that cognitive flexibility—the tendency to adapt thinking and switch perspectives in processing information and solving problems (Dane, 2010; Kiss et al., 2020)—serves as one of the key intermediating mechanisms through which knowledge counterflow facilitates older employee creativity.

**Figure 1:**
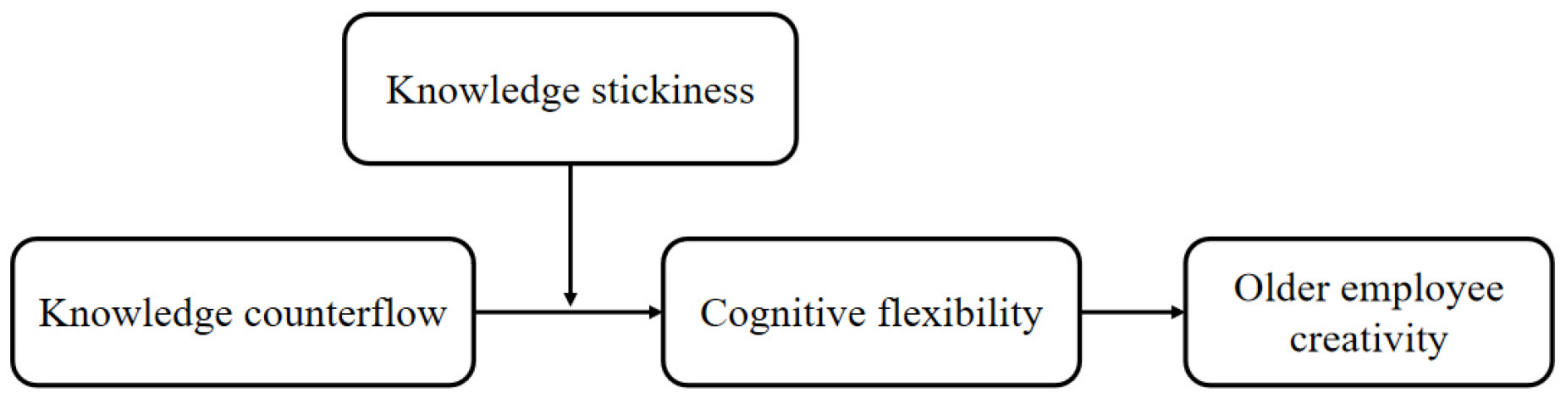
The proposed research model.

However, knowledge counterflow may not necessarily result in a higher level of cognitive flexibility and older employee creativity. As argued before, older employees need to be aware of the new frameworks of thinking. Therefore, we argue that the positive influence depends highly on knowledge stickiness, i.e., the difficulty for the older employees to understand the transferred knowledge (Huan et al., 2017). Under the condition of a low level of knowledge stickiness, the older employees can get a clear understanding of the new frameworks. They can thus readily adapt their thinking to the new frameworks, i.e., increasing cognitive flexibility, and thus be more creative. By contrast, when knowledge stickiness is high, the older employees cannot understand the new knowledge and may not be able to get a clear sense of the new ways of thinking. In this situation, they tend to process the information and knowledge within their own established framework (Wen et al., 2023); cognitive flexibility and creativity will thus not be facilitated. To test the logic, in this study, we set out to examine the moderating role of knowledge stickiness (see in Figure 1).

Our study makes three main contributions. First, it poses challenge to the stereotype that older employees are less creative. This study suggest that older employees have the capabilities to be creative, highlighting the possibility of harnessing their creative potential. Second, our study advances the current understanding of knowledge transfer. Prior research has primarily focused on knowledge transfer from the older to younger employees. However, the rapid advancement of new technologies in the past decade makes it likely that older employees need to learn from their younger colleagues (Pfrombeck et al., 2024). Our study thus extends the research into knowledge counterflow to understand how this type of knowledge transfer affects older employee creativity. Third, we help clarify the theoretical mechanism of how knowledge counterflow stimulates older employee creativity. We propose that knowledge counterflow makes older employees cognitively more flexible and leads them to exhibit a higher level of creativity, and that knowledge stickiness serves as a critical boundary condition.

## Theoretical background and hypotheses development

### Knowledge counterflow

Pfrombeck et al. (2024) pioneered the examination of “older workers' knowledge seeking from younger coworkers,” emphasizing an understanding of intergenerational knowledge acquisition from the perspective of the knowledge receiver's (i.e., older employees') behavioral motives. In contrast, “knowledge counterflow” is a more inclusive construct that encompasses not only older employees' active seeking behaviors but also younger employees' active knowledge transmission, as well as the bidirectional interactive processes that may occur between them (Fasbender et al., 2021). In other words, knowledge counterflow emphasizes the directionality of knowledge flow from the younger generation to the older generation, rather than the behavioral characteristics of specific actors. This conceptual choice is supported by two theoretical justifications. First, it maintains conceptual continuity with the “knowledge flow” tradition in the knowledge management literature (Szulanski, 1996). Second, it more fully captures the multiple interaction patterns that may exist in intergenerational knowledge transfer, rather than being confined to the receiver's unilateral information acquisition behavior. At the same time, knowledge counterflow should not be conflated with reverse mentoring. Reverse mentoring refers to a structured human resource development program formally established by organizations, in which younger employees serve as mentors and older employees as mentees (Chaudhuri and Ghosh, 2012; Marcinkus Murphy, 2012)—an institutionalized intervention tool. Knowledge counterflow, by contrast, is a broader phenomenological concept that can occur not only within formal reverse mentoring programs but also emerge organically through informal daily interactions, cross-generational project collaboration, social media exchanges, and other channels (Rudolph et al., 2018). In other words, reverse mentoring represents one possible formal mechanism through which organizations can institutionalize knowledge counterflow, but the phenomenon itself is not confined to—nor synonymous with—any particular programmatic form.

### Older employee creativity

As the mean age of the workforce is rising in most industrial countries, research attention has been increasingly paid to understand how older employees contribute organizational productivity (Ng and Feldman, 2008; Sturman, 2003). Despite of the widely-held negative stereotype that older employees are less creative, empirical findings do not support such a belief (e.g., Ng and Feldman, 2013). Actually, anecdotal evidence suggested that people of older age have great creative potential. Therefore, in this study, we seek to understand how to encourage older employee creativity. Creativity embodies novel ideas or thought processes that diverge significantly from the organization's current practices (Madjar et al., 2011). It places a stronger emphasis on changing the status quo, thus potentially yielding greater prospective benefits, albeit accompanied by elevated risks and uncertainties (Gilson and Madjar, 2011; Madjar et al., 2011). Creativity encompasses the pivotal processes of generating original ideas and evaluating their potential for implementation (Perry-Smith and Mannucci, 2017). Creative ideas represent a departure from the established perceptions, thus creating novel possibilities (Gilson and Madjar, 2011).

In order to generate creative ideas, older employees need to get involved with diverse information, depart from the conventional patterns of thinking, and enhance their ability to flexibly transition between tasks to acquire and integrate more information, thereby sparking new associations (Gilson and Madjar, 2011; Mumford and Gustafson, 1988). The processes require a larger repository of information and the recombination of diverse knowledge into new associations (Madjar et al., 2011; Zhang L. et al., 2023; Zhang Y. et al., 2023). We thus propose that knowledge counterflow can augment older employees' information resources by providing non-redundant information, as the scope and structure of knowledge held by employees of different generations can be complementary (Burmeister et al., 2020).

Why Focus on Knowledge Counterflow (Younger-to-Older) Rather Than the Reverse Direction? One might reasonably ask whether the proposed model would also apply to younger employees seeking knowledge from older coworkers. We argue that the mechanisms are asymmetrical for two theoretical reasons. First, younger and older employees face fundamentally different creative bottlenecks. Younger employees typically possess higher fluid intelligence and more flexible cognitive structures (Murmann et al., 2023), but lack the deep domain expertise and contextual knowledge accumulated over years of experience (Dane, 2010). Their creative challenge lies primarily in knowledge acquisition and depth, not cognitive flexibility. By contrast, older employees possess rich domain expertise but face the risk of cognitive entrenchment—a rigidity that stems from over-reliance on established knowledge frameworks (Gerpott et al., 2017). Thus, the same external knowledge input produces distinct effects depending on the recipient's cognitive baseline. Second, the content of knowledge flowing in each direction differs qualitatively. Younger employees tend to possess knowledge that is more novel, technology-oriented, and misaligned with older employees' existing frameworks (Fasbender and Gerpott, 2022)—precisely the type of incongruent input that disrupts entrenched thinking. In contrast, the knowledge older employees typically share with younger coworkers is more experience-based and domain-deepening, which is more likely to enhance incremental refinement rather than fundamental cognitive restructuring (Gilson and Madjar, 2011). Therefore, the cognitive flexibility mechanism we propose is theoretically specific to the context of older employees receiving knowledge from younger coworkers, rather than a general model applicable to all intergenerational knowledge transfers.

Knowledge counterflow refers to the process of knowledge transfer from younger senders to older recipients (Fasbender and Gerpott, 2022; Wang et al., 2023; Pfrombeck et al., 2024). Younger employees get to knowledge resources around cutting-edge technology and science, along with related abstract and conceptual knowledge, and the ability to navigate workplace insecurities, dynamic environments, and diversity (Ebrahimi et al., 2008; Li et al., 2021). Older employees, in contrast, typically have higher domain-specific expertise and experience (Fasbender et al., 2021), and can therefore benefit from younger employees' knowledge reserves (Kollmann et al., 2020). In terms of their cognitive structures, younger employees exhibit higher fluid intelligence capabilities and more flexible knowledge structures, enabling them to be effective in dynamic, ambiguous, and complex environments (Murmann et al., 2023; Zhang L. et al., 2023; Zhang Y. et al., 2023). Meanwhile, older employees can also apply their previous experience to new problems (Xie et al., 2023). The breadth and diversity of the information resources resulting from differences in knowledge and cognitive structures can effectively foster creativity (Li et al., 2021). Therefore, we hypothesize:

*Hypothesis 1: knowledge counterflow is positively related to older employee creativity*.

### Cognitive flexibility as the intermediating mechanism

According to cognitive flexibility theory, by learning a new domain of knowledge, people not only acquire information, but also get new perspectives and frameworks. The awareness of alternatives and choices can help enhance cognitive flexibility (Martin and Rubin, 1995), which lays the foundation for innovation and creative problem solving (Dajani and Uddin, 2015; Dane, 2010). The theory emphasizes the importance of cross-contextual learning (Spiro, 1988), and stresses that multi-perspective information helps facilitate cognitive flexibility. We argue that knowledge counterflow can give older employees a learning opportunity that is intellectually diverse, enhancing their cognitive flexibility. As mentioned, the scope and structure of the knowledge held by employees of different generations differs greatly. In this sense, knowledge counterflow can increase intergenerational learning and stimulating the combination of new domains of knowledge (Carnevale et al., 2021). Knowledge counterflow thus extends older employees' existing cognitive frameworks by introducing new and diverse information (Braem and Egner, 2018; Rui and Ju, 2022). This can lead them to reevaluate and update their knowledge frameworks and perspectives. Their structures of knowledge may be reconfigured (Locke et al., 2008; Maitlis and Sonenshein, 2010), helping inspire creative insights (Beier et al., 2022; Scheuer et al., 2023) and increase the likelihood that new associations and connections will be formed as more diverse information is received (Mark et al., 2020; Simoens et al., 2024). Thus, knowledge counterflow can help broaden the associative networks of older employees and enhance their cognitive flexibility (Wen et al., 2023).

From the perspective of cognitive aging, cognitive flexibility is particularly critical for older employees. Unlike younger employees, whose cognitive structures remain relatively malleable, older employees often face the risk of cognitive entrenchment due to their long-term reliance on accumulated experience and established knowledge frameworks (Dane, 2010). Although their rich domain expertise provides a solid foundation for creativity, it may also inadvertently constrain their ability to think beyond existing paradigms (Gerpott et al., 2017). Therefore, for older employees, the key to stimulating creativity lies not in acquiring more knowledge *per se*, but in restructuring their cognitive frameworks to maintain flexibility (Ng and Feldman, 2008). Knowledge counterflow, as a source of novel and non-redundant information, offers a promising pathway to achieve this cognitive restructuring. Creativity requires breaking out of existing cognitive frameworks and generating new ideas. It often involves the integration of diverse information and knowledge to make new associations (Madjar et al., 2011; Zhang L. et al., 2023; Zhang Y. et al., 2023). Older employees have typically accumulated deep industry knowledge and rely on familiarity and their previous experiences when solving problems (Dane, 2010; Zhu et al., 2023). Dane (2010) suggests that long-term reliance on prior knowledge and experience can lead to fixed pattern of thinking, making it difficult to generate new ideas. Thus, although they can effectively solve the similar problems, it is difficult for them to deal with new challenges (Rui and Ju, 2022; Scheuer et al., 2023; Xia et al., 2024). Therefore, stimulating older employee creativity requires not only acquire new knowledge, but also broaden cognitive boundaries and increased cognitive flexibility from multiple perspectives and levels (Dajani and Uddin, 2015; Uddin, 2021).

Cognitive flexibility can help facilitate older employee creativity. First, it can help older employees counteract the age-related decline of fluid intelligence which relates to logical thinking, problem solving, and information processing (Xia et al., 2024), and enhances their information processing abilities (Ng and Feldman, 2013). By developing cognitive flexibility, older employees can adapt to new environments and assimilate and apply new information more effectively, thereby remaining cognitively active and improving their ability to solve problems (Guillén and Kunze, 2019). Second, cognitive flexibility allows older employees to quickly adapt to changes in technology and working methods, to merge old and new knowledge, and to acquire new skills (Tams and Dulipovici, 2022). Such a flexibility also helps them to combine different information or concepts to create new solutions (Volmer et al., 2019). In addition, cognitive flexibility in older employees enables them to explore unknown paths and experiment with new possibilities when faced with complex and uncertain situations (Beier et al., 2022; Hüber et al., 2022; Kelly et al., 2014). Thus, the knowledge and experience accumulated over time, combined with the development of cognitive flexibility, encourages them to push the boundaries of their knowledge and skills and explore new areas. Thus, cognitive flexibility not only enhances older employees' adaptive capacity, but also serves as a key driver to be creative (Rebok et al., 2014). Taken together, this leads to our second hypothesis.

*Hypothesis 2: cognitive flexibility mediates the relationship between knowledge counterflow and older employee creativity*.

### Moderating role of knowledge stickiness

In this study, we further argue that the positive effect of knowledge counterflow on older employee creativity through cognitive flexibility may be moderated by knowledge stickiness. Knowledge stickiness refers to the difficulty in understanding the knowledge transferred (Li and Hsieh, 2009; Riusala and Smale, 2007; Szulanski et al., 2016). Importantly, knowledge stickiness is not a unidimensional construct. Beyond the recipient's comprehension difficulty, prior research has identified multiple sources of stickiness, including characteristics of the knowledge itself (e.g., tacitness, complexity, causal ambiguity; Simonin, 1999; Szulanski, 1996), characteristics of the knowledge sender (e.g., ability to articulate, willingness to share; Szulanski et al., 2004), communication barriers between generational groups (Schmidt and Muehlfeld, 2017), and organizational support systems that may unintentionally impede knowledge flow (Riusala and Smale, 2007). In this study, we focus on the recipient-perceived stickiness—i.e., older employees' subjective experience of difficulty in understanding and absorbing the knowledge transferred from younger colleagues. We adopt this focus because perceived stickiness is the most proximal psychological barrier to cognitive flexibility: regardless of whether the difficulty originates from the knowledge itself, the sender, or the organizational context, it is the older employees' subjective experience of that difficulty that directly shapes their cognitive engagement with the new knowledge. When older employees find the knowledge difficult to comprehend, they are likely to disengage from deep cognitive processing and fall back on existing frameworks, thereby precluding the enhancement of cognitive flexibility. Extensive unabsoable information may precipitate cognitive closure in older employees. Processing complex and unfamiliar information and knowledge consumes excessive cognitive resources (Dajani and Uddin, 2015; Uddin, 2021), leaving fewer resources available for integrating the knowledge into the current structure. To mitigate the further depletion of these resources, older employees are likely to simplify their approach to information processing (Wen et al., 2023), which can potentially suppress their cognitive flexibility (Koch et al., 2018). In essence, knowledge stickiness increases the cost of knowledge transfer and hinders knowledge acquisition (Szulanski, 1996; Szulanski et al., 2016). Knowledge stickiness increase the cost of knowledge transfer and understanding, thus decreasing the efficiency of information processing (Szulanski, 1996).

A lower level of knowledge stickiness enables knowledge to be transferred smoothly and understood easily (Schmidt and Muehlfeld, 2017). In this sense, older employees can absorb the knowledge from younger employees and be aware of the new framework of thinking, thereby facilitating cognitive flexibility. Creativity, which relates to the ability to extend the existing cognitive boundaries and think outside the box to solve problems, is affected by the absorption of heterogeneous information (Liu et al., 2016). Therefore, in the context of knowledge counterflow, the level of knowledge stickiness may impact the cognitive flexibility of older employees (Uddin, 2021). Therefore, we propose the following hypotheses:

*Hypothesis 3: Knowledge stickiness moderates the relationship between knowledge counterflow and cognitive flexibility, such that when knowledge stickiness is low, the positive effect of knowledge counterflow on cognitive flexibility is more pronounced*.

*Hypothesis 4: Knowledge stickiness moderates the mediating effect of cognitive flexibility in the relationship between knowledge counterflow and older employee creativity; when knowledge stickiness is low, the positive effect of knowledge counterflow on older employee creativity through cognitive flexibility is more pronounced*.

## Method

### Respondents and procedure

To test the hypotheses proposed in this study, we conducted a two-wave questionnaire survey among R&D personnel from three high-tech enterprises located in western and eastern China, between February and April 2025. R&D departments were selected as the research setting because creativity is a core performance indicator for such positions. Enterprises were recruited through the following process: we first obtained enterprise directories from local science park management committees, then sent invitation emails and made phone calls to invite eligible enterprises, and finally signed participation agreements with those that agreed to participate.

Employees and their supervisors were recruited as follows: the human resources department of each participating enterprise distributed invitation letters via internal emails to eligible R&D employees, clearly stating the research purpose, voluntary participation principles, and anonymity guarantees. Interested employees were required to sign informed consent forms. To ensure the accuracy of data matching, each participant was assigned a unique research code (composed of company code + individual serial number, e.g., A001), which was independently generated and kept sealed by the research team. The HR departments of the participating enterprises had no access to the correspondence between codes and names.

To alleviate the potential influence of common method bias, this study adopted a multi-source, multi-wave data collection design. Specifically, at Time 1 (T1), older employees (aged ≥40) self-reported their demographic information (age, gender, education level, organizational tenure), knowledge counterflow, and knowledge stickiness. Questionnaires were distributed and collected on-site by two trained master's students. A total of 507 questionnaires were distributed, and 424 valid questionnaires were returned, yielding a response rate of 83.6%. At Time 2 (T2)-−4 weeks after T1 (i.e., April 2025)—the research team distributed the second-wave questionnaires to the 424 employees who had completed the T1 survey and their direct supervisors. Employees self-reported their cognitive flexibility, while supervisors rated their subordinates' creativity (each supervisor evaluated 1–2 employees). At T2, 385 employee questionnaires were returned (response rate: 90.8%) and 362 supervisor questionnaires were returned (response rate: 85.4%). The research team longitudinally matched the employee self-report questionnaires from T1 and T2 using the unique research codes, and then matched these with the supervisor evaluation data across sources. After matching, we obtained 323 valid paired questionnaires (overall effective response rate: 76.2%), corresponding to 323 older employees and 189 direct supervisors (i.e., an average of approximately 1.71 employees evaluated by each supervisor).

In our final sample, we used the age of 40 as the cutoff point for defining older employees, based on three main considerations. First, for creative jobs such as those in R&D units, this age represents an important psychological milestone in one's career (Li et al., 2021). Second, legislation protecting employees from age discrimination in many countries also often uses this age as the standard (e.g., the Age Discrimination in Employment Act in the United States). Third, this cutoff point has been adopted in previous empirical studies (e.g., Cloostermans et al., 2015; Hansson et al., 1997). The demographic characteristics of our final sample are as follows: males accounted for 38.4% and females for 61.6%; in terms of education level, 83.7% held a master's degree or above; regarding organizational tenure, 57.9% had more than 15 years of work experience; in terms of age distribution, 51.7% were in the 40–45 age group and 48.3% were aged above 45.

### Measures

The measures used in this study were originally developed in English and translated into Chinese following the translation–back-translation approach.

### Knowledge counterflow

Knowledge counterflow was assessed using four items adapted from the seminal works of Pfrombeck et al. (2024) and Wilkesmann et al. (2009). The respondents were asked to report their level of agreement with the statements on 7-point scales (1 = strongly disagree; 7 = strongly agree). An example is, “I turn to my younger colleagues for advice regarding new technologies so that I learn them” (α = 0.90).

### Cognitive flexibility

The 12 items for assessing cognitive flexibility were adapted from Martin and Rubin (1995). The respondents were asked to report their level of agreement with these statements on a 7-point scale (1 = strongly disagree; 7 = strongly agree; α = 0.88). An example item is “I am willing to listen and consider alternatives for handling a problem.” The Cronbach's alpha was 0.893.

### Knowledge stickiness

We used four items from Simonin (1999) in our assessment of knowledge stickiness. The respondents were asked to rate their agreement with these statements on a 7-point scale (1 = strongly disagree; 7 = strongly agree). An example item is “The knowledge passed on to me by my younger colleagues is hard to express clearly and explicitly in written form, such as manuals, work instructions, and product design drawings” (α = 0.82).

### Older employee creativity

We asked the direct supervisors of older employees to assess their creativity with four items adapted from Madjar et al. (2011) and Baer (2012). Supervisors were asked to rate the extent to which they agree that the employee engages in the activities in the statement at a 7-point scale (1 = strongly disagree; 7 = strongly agree). An example item is “this employee often come up with many creative ideas.” The Cronbach's alpha was 0.842.

### Control variables

In this study, we controlled for four demographic variables, i.e., the age of employees and their organizational tenure, gender, and level of education. We included age as a variable to explore its potential effects on the focal process and outcome variables. We controlled for the demographic characteristic of gender to examine any possible gender-related differences in social interactions. Gender was coded as a dummy variable (0= female, 1 = male). According to the literature, gender and age can influence an individual's social behavior (Burmeister et al., 2022). Education level and organizational tenure were also included as control variables, as these are important indicators of firm-specific knowledge (Harvey, 2012). Organizational tenure also influences employees' learning experiences. A longer tenure may enhance employees' feelings of attachment to their organizations, which has been identified as a key factor in motivating employees to continue working (Mathieu and Zajac, 1990).

## Results

### Descriptive statistics

To examine whether the key variables are discriminable, we conducted confirmatory factor analyses. As shown in Table 1, the four-factor model, i.e., knowledge counterflow, cognitive flexibility, older employee creativity, and knowledge stickiness, fit the data (χ^2^/df = 1.268, RMSEA = 0.029; NFI = 0.944; IFI = 0.988; TLI = 0.986) much better than the alternative models which combine knowledge counterflow with cognitive flexibility (Δχ^2^ = 783.926, *p* < 0.05) and that combining knowledge counterflow with cognitive flexibility and older employee creativity (Δχ^2^ = 1399.948, *p* < 0.05). The results suggested a good construct validity for the variables, supporting our further analyses.

**Table 1:** The results of confirmatory factor analyses.

Models	*χ^2^/*df	*RMSEA*	*NFI*	*IFI*	*TLI*
1 Four-factor measurement model	1.268	0.029	0.944	0.988	0.986
2 Combine knowledge counterflow and cognitive flexibility	4.401	0.103	0.806	0.843	0.825
3 Combine knowledge counterflow, cognitive flexibility and knowledge stickiness	6.821	0.134	0.696	0.729	0.700
4 One-factor model	9.780	0.165	0.563	0.589	0.548

NFI is the normed fit index. IFI is the incremental fit index. TLI is the Tucker-Lewis coefficient, also known as non-normed fit index (NNFI). RMSEA is the root mean square error of approximation.

Table 2 provides the descriptive statistics and correlations of the variables. Knowledge counterflow was positively correlated with cognitive flexibility (*r* = 0.323, *p* < 0.01), and with older employee creativity (*r* = 0.436, *p* < 0.01). ^*^p < 0.05, ^**^p < 0.01. Meanwhile, cognitive flexibility was positively related with older employee creativity (*r* = 0.440, *p* < 0.01). These results offer preliminary support for the proposed relationships.

**Table 2:** Mean, standard deviations, and correlations.

Variables	Mean	SD	1	2	3	4	5	6	7
1 Age	1.57	0.94							
2 Gender	0.58	0.49	−0.188^**^						
3 Education	1.83	0.41	−0.314^**^	0.128^**^					
4 Organizational tenure	3.59	1.39	0.549^**^	−0.030	−0.270^**^				
5 Knowledge counterflow	4.08	1.30	−0.074	0.056	0.084	−0.048			
6 Cognitive flexibility	4.15	1.11	−0.008	−0.052	0.045	−0.037	0.323^**^		
7 Older employee creativity	3.89	1.34	−0.050	−0.008	0.066	−0.087	0.436^**^	0.440^**^	
8 Knowledge stickiness	4.11	1.22	0.021	0.001	0.057	0.009	−0.078	−0.080	−0.026

*N* = 323. Coefficient alpha reliabilities are in parentheses along the diagonal. Age is coded as 2 = above 45, 1 = 40 to 45. Gender is coded as 0 = female,1 = male. Education is coded as 2 = master's degree or above; 1 = no master's degree. Organizational tenure is coded as 1 = more than 15 years, 0 = fewer than 15 years. ^*^p < 0.05. ^**^p < 0.01.

### Further validation of discriminant validity

In addition to confirmatory factor analysis (CFA), we further calculated the average variance extracted (AVE) and composite reliability (CR) for each construct to more rigorously assess discriminant validity. As shown in Table 3, the AVE values for all constructs exceeded the recommended threshold of 0.50 (Fornell and Larcker, 1981), and the CR values all exceeded 0.70, indicating good convergent validity. Moreover, the square root of the AVE for each construct was greater than its correlations with other constructs, further supporting discriminant validity.

**Table 3:** Construct reliability and validity.

Construct	Cronbach's α	CR	AVE	Square root of AVE
Knowledge counterflow	0.9	0.902	0.65	0.806
Cognitive flexibility	0.88	0.884	0.561	0.749
Knowledge stickiness	0.82	0.825	0.542	0.736
Older employee creativity	0.84	0.845	0.578	0.76

CR, composite reliability; AVE, average variance extracted. The square root of AVE for each construct is greater than its correlations with other constructs, supporting discriminant validity.

### Common method bias test

This study controlled for the potential risk of common method bias procedurally through a multi-source, multi-wave research design. Concurrently, we conducted a statistical test using Harman's single-factor test: all measurement items were entered into an unrotated exploratory factor analysis. The results showed that the first factor explained only 26.8% of the total variance, well-below the 40% threshold recommended by Podsakoff et al. (2003). Therefore, common method bias is unlikely to threaten the validity of our findings.

### Cluster-robust standard errors

Considering the nested structure of the data at the supervisor level (189 supervisors evaluating 323 employees), and despite the relatively small average number of employees evaluated per supervisor (1.71), we employed cluster-robust standard errors to re-estimate all hypothesized paths (Colin Cameron and Miller, 2015), in order to control for potential estimation bias in standard errors arising from unobserved heterogeneity at the supervisor level. As shown in Table 4, the significance of all paths after clustering adjustment remained consistent with the main analysis, indicating that our findings are robust to the nested data structure.

**Table 4:** Results of cluster-robust standard errors analysis.

Path	*b* (main analysis)	*p* (main analysis)	*p* (cluster-adjusted)	Significance changed?
Knowledge counterflow → Creativity (H1)	0.444	< 0.001	< 0.001	No
Knowledge counterflow → Cognitive flexibility	0.275	< 0.001	< 0.001	No
Cognitive flexibility → Creativity	0.351	< 0.001	< 0.001	No
Knowledge counterflow × Knowledge stickiness → Cognitive flexibility (H3)	−0.146	< 0.01	< 0.01	No

Cluster-robust standard errors were estimated with 189 supervisors nested across 323 employees. All paths remained significant after clustering adjustment, indicating that the results are robust to the nested data structure.

### Tests of hypotheses

Hypothesis 1 suggests that knowledge counterflow facilitates older employee creativity. To test this hypothesis, we first regressed older employee creativity on the control variables and the independent variable (i.e., knowledge counterflow). The results, shown in Table 5, showed that knowledge counterflow is positively related to older employee creativity (*b*= 0.444, *p* < 0.001),^*^p < 0.05, ^**^p < 0.01, ^***^p < 0.001, supporting Hypothesis 1.

**Table 5:** Process regression analyses for cognitive flexibility and older employee creativity.

Variables	Cognitive flexibility				Older employee creativity			
	Model 1		Model 2		Model 3		Model 3	
	*b*	SE	*b*	SE	*b*	SE	*b*	SE
Age	0.041	0.090	0.051	0.089	0.037	0.104	0.030	0.095
Gender	−0.163	0.124	−0.159	0.121	−0.101	0.142	−0.040	0.131
Education	0.080	0.144	0.068	0.142	0.082	0.165	0.021	0.152
Organizational tenure	−0.029	0.052	−0.041	0.052	−0.076	0.060	−0.080	0.055
Knowledge counterflow	0.275^***^	0.045	0.274^***^	0.044	0.444^***^	0.048	0.353^***^	0.051
Cognitive flexibility							0.351^***^	0.061
Knowledge stickiness			−0.062	0.047			0.014	0.051
Knowledge counterflow × Knowledge stickiness			−0.146^***^	0.040			−0.180^***^	0.044
*R^2^*	0.111		0.151		0.196		0.331	

*N* = 323. ^*^p < 0.05; ^**^p < 0.01; ^***^p < 0.001.

Hypothesis 2 further suggests that cognitive flexibility mediates the positive relationship between knowledge counterflow and older employee creativity. We used the three step approach to examine this hypothesis (Baron and Kenny, 1986). The mediator variable, i.e., cognitive flexibility, was regressed on the control variables and the independent variable, i.e., knowledge counterflow. Results showed (see in Table 5) that knowledge counterflow had a significantly positive effect on cognitive flexibility (*b* = 0.275, *p* < 0.001). Older employee creativity was then regressed on the control variables, knowledge counterflow, and cognitive flexibility. Results showed a significantly positive relationship between the mediator variable of cognitive flexibility and the dependent variable of older employee creativity (*b* = 0.351, *p* < 0.001). The empirical evidence and the findings for Hypothesis 1 together indicate that cognitive flexibility mediated the relationship between knowledge counterflow and older employee creativity. To further examine the indirect effect, we used the *PROCESS* macro in SPSS to examine the indirect relationship between knowledge counterflow and older employee creativity via cognitive flexibility. Table 6 indicates that the size of the indirect effect was .096, 95% confidence interval (CI) (0.054, 0.150), thus further confirming a significant indirect effect and thus supporting Hypothesis 2.

**Table 6:** Results of bootstrapping tests for indirect effects.

Conditions	Effect size	SE	95%CI	
			Lower	Upper
Direct effect	0.353	0.051	0.254	0.452
Direct effect				
High knowledge stickiness	0.134	0.070	−0.004	0.272
Low knowledge stickiness	0.572	0.076	0.422	0.722
Indirect effect	0.096	0.025	0.054	0.150
Indirect effect				
High knowledge stickiness	0.034	0.027	−0.019	0.089
Low knowledge stickiness	0.159	0.037	0.096	0.239
Difference	−0.125	0.042	−0.217	−0.055

The bootstrapping analysis was conducted with 20,000 repetitions.

**Figure 2:**
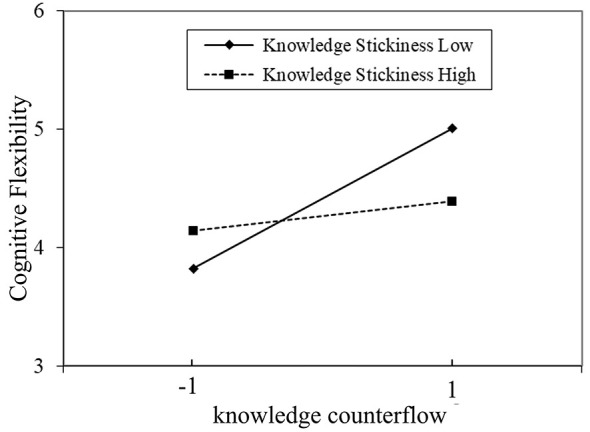
Moderating effect of knowledge stickiness on the relationship between knowledge counterflow and cognitive flexibility.

Hypothesis 3 proposes that knowledge stickiness has a moderating effect on the relationship between knowledge counterflow and cognitive flexibility. To test this hypothesis, we examined the effect of cognitive flexibility on the independent variable of knowledge counterflow, the moderator variable of knowledge stickiness, and their interaction terms. The analysis results, presented in Tables 5, 6, indicate that the interaction term between knowledge counterflow and knowledge stickiness significantly affected older employee creativity (*b* = −0.146, *p* < 0.01). Following the recommendations of Cohen et al. (2003), we plotted the interaction pattern in Figure 2. The simple slopes test results show that the effect was significant at low levels of knowledge stickiness (*b* = 0.453, *p* < 0.01) and not significant at high levels (*b* = 0.096, *p* = 0.148).

We found that under conditions of high knowledge stickiness (+1 SD), the size of the indirect effect was.034, with a 95% CI of (−0.019, 0.089). However, under the condition of a low level of knowledge stickiness (−1 SD), the effect size was.159, with a 95% CI of (0.096, 0.239). The difference in effects under these two conditions was significant [*d* = −0.125, 95% CI (−0.217, −0.055)]. These results, summarized in Table 6, also provide support for Hypothesis 4, which was therefore confirmed by the overall findings.

## Discussion

In this study, we propose that knowledge counterflow can be a factor that facilitate older employee creativity and investigate its impact mechanism and boundary conditions. Our multi-timepoint questionnaire data from 323 direct leader-employee pairings revealed various key findings. First, the knowledge counterflow from younger to older employees can encourage older employee creativity. Second, cognitive flexibility plays a crucial mediating role in this process. Knowledge counterflow increases older employee creativity by facilitating cognitive flexibility. Third, knowledge stickiness moderates the process. Under the condition of low knowledge stickiness, the effect of knowledge counterflow on cognitive flexibility and its subsequent enhancement of older employee creativity is more pronounced. This suggests that lower levels of knowledge stickiness facilitate the flow and absorption of knowledge, thereby increasing its positive impact on older employee creativity. In addition, the moderating effect of knowledge stickiness on the direct relationship between knowledge counterflow and older employee creativity indicates that reducing the barriers to knowledge flow can significantly improve older employee creativity. The findings provide profound theoretical and practical implications.

One noteworthy finding of this study is that when knowledge stickiness is high, the positive effect of knowledge counterflow on cognitive flexibility becomes non-significant (b =0.096, *p* = 0.148). This implies that not all forms of knowledge counterflow are equally effective in activating older employee creativity—its effectiveness hinges on whether the knowledge can be smoothly understood and absorbed. From the perspective of cognitive resource theory, this finding carries important theoretical implications. When the knowledge conveyed by younger employees is highly sticky (e.g., obscure, overly abstract, or poorly connected to the existing knowledge system), older employees are required to expend substantial cognitive resources on basic information decoding and comprehension, rather than on higher-order creative knowledge integration (Dajani and Uddin, 2015; Uddin, 2021). Under such circumstances, older employees may adopt a “cognitive economizing” strategy—processing new information within their existing cognitive frameworks while minimizing the additional cognitive effort needed to restructure their knowledge architecture (Wen et al., 2023). Although this strategy is cognitively efficient in the short term, it comes at the cost of failing to internalize new mental frameworks, thereby precluding any substantive enhancement of cognitive flexibility. What merits further consideration is that knowledge transfer under high-stickiness conditions effectively constitutes a state of “ineffective input”—novel information is received, but fails to be encoded and integrated into existing cognitive structures, and thus cannot trigger the activation of creativity. This finding suggests that in examining the relationship between knowledge transfer and creativity, researchers should not focus solely on the “quantity” of knowledge flow (i.e., the frequency or breadth of knowledge transfer), but should also attend to its “quality” (i.e., whether the knowledge recipient truly understands and internalizes the new knowledge). This insight echoes Szulanski's (1996) classic argument that “knowledge stickiness is essentially the friction of knowledge transfer”—when friction is excessive, even the appearance of knowledge flow may produce effects approaching zero.

### Theoretical implications

First, this study challenges the stereotype that older employees are less creative (Dane, 2010; Zhu et al., 2023). Our findings indicate that those older employees are capable of engaging in creative activities. Their rich professional knowledge provides a solid foundation for creativity. Nevertheless, the experiences may also lead them to rely more on established frameworks even when dealing with new challenges. Such a reliance can sometimes result in cognitive rigidity, thus limiting creative thinking (Mansour and Tremblay, 2019). Therefore, enhancing older employee creativity requires not only leveraging their accumulated knowledge, but also the expansion of cognitive boundaries through taking multiple perspectives, as this can enhance their cognitive flexibility (Wen et al., 2023). Knowledge counterflow, i.e., absorbing novel and non-redundant knowledge, can help mitigate the potential decline in cognitive ability associated with aging, thus providing the necessary foundation for innovative thinking (Perry-Smith, 2014; Pfrombeck et al., 2024). Our research not only extends the understanding of the relationship between age and creativity, but also highlights the value of older employees in promoting organizational innovation, offering new insights for human resource management, especially when designing age-diversity strategies and training programs.

Second, this study advances our understanding of knowledge transfer. Previous research has focused on the transfer of knowledge from older to younger employees, emphasizing the inheritance of experience and skills (Fasbender and Gerpott, 2022; Huan et al., 2017; Sheng et al., 2013). However, our research reveals a more complex interactive process, in which knowledge transfer is not unidirectional. The new perspectives of younger employees and their up-to-date knowledge can also influence older employees, fostering bidirectional knowledge flow. This finding enriches the theoretical landscape of knowledge management, suggesting that organizations should adopt more flexible and inclusive knowledge transfer mechanisms to promote innovation and learning.

Third, our study theoretically establishes the process through which knowledge transfer stimulates creativity, and identifies cognitive flexibility as a mediating explanatory mechanism, with knowledge stickiness as a boundary condition. We found that knowledge transfer can enhance creativity by increasing the flexibility of individuals' cognition, and that this process is moderated by knowledge stickiness. If knowledge is easier to understand and absorb and thus has a lower level of stickiness, knowledge transfer will have a greater positive impact on cognitive flexibility, thereby facilitating creativity. This provides a foundation for the design of effective knowledge management strategies, in which knowledge transfer and innovation capabilities can be enhanced by reducing knowledge stickiness.

Furthermore, our findings may primarily speak to incremental creativity—the refinement and extension of existing ideas—rather than radical creativity. This distinction is theoretically meaningful because older employees' deep domain expertise, accumulated over years of experience, makes them particularly well-suited for incremental innovation, which relies on the recombination of existing knowledge within familiar domains (Madjar et al., 2011; Katila and Ahuja, 2002). Knowledge counterflow, by providing non-redundant and novel knowledge inputs from younger colleagues, may help older employees recombine their existing knowledge in novel ways—the very essence of incremental creativity. At the same time, whether knowledge counterflow also stimulates radical creativity—which requires more fundamental cognitive frame-breaking and the generation of entirely new paradigms—remains an open question for future research. We encourage scholars to adopt specialized creativity measures (e.g., Madjar et al., 2011; Zhang L. et al., 2023; Zhang Y. et al., 2023) to empirically disentangle the differential effects of knowledge counterflow on these two creativity forms, and to explore whether additional mechanisms (e.g., psychological safety, risk tolerance) are required for radical creativity to emerge.

### Practical implications

First, recognize the creative potential of older employees and break down age stereotypes. Our findings indicate that older employees possess significant innovative potential. Managers should abandon the traditional bias that “older equals conservative” and view older employees as a positive force for innovation rather than as a burden to be “managed.” Specifically, organizations should include older employees in the core teams of innovation projects, rather than marginalizing them to supporting roles. In innovation incentive systems, organizations should ensure that older employees enjoy the same recognition and reward opportunities for innovation as their younger counterparts.

Second, enhance the effectiveness of knowledge counterflow by reducing knowledge stickiness. A core finding of this study—that knowledge stickiness significantly weakens the positive effects of knowledge counterflow—suggests that managers should not merely settle for superficial arrangements such as “having younger employees teach older colleagues.” Instead, they should focus on reducing barriers in the knowledge transfer process. Specific strategies include: Knowledge visualization: encouraging younger employees to convert technical knowledge into flowcharts, video tutorials, and standardized documentation to reduce the level of abstraction and tacitness of knowledge (Huan et al., 2017). Peer learning partnerships: establishing cross-generational “learning partner” relationships (rather than traditional “mentor-mentee” relationships) to reduce the psychological pressure and face-saving concerns arising from formal hierarchical structures, allowing older employees to learn new knowledge in a more egalitarian atmosphere. Regular “knowledge marketplaces”: hosting monthly informal technology sharing sessions where younger employees share cutting-edge technologies and new methods through “show-and-tell” rather than formal “teaching,” lowering the psychological threshold for older employees to admit knowledge gaps.

Third, enhance older employees' cognitive flexibility through systematic interventions. Our findings identify cognitive flexibility as the key mediating mechanism through which knowledge counterflow promotes creativity, implying that organizations should not only focus on the transmission of knowledge content but also attend to the shaping of employees' cognitive patterns. The following measures are recommended: cross-generational project rotation: arranging for older employees to periodically participate in new projects led or core-participated by younger employees, introducing new thinking paradigms and problem-solving frameworks through immersive exposure. Institutionalizing “reverse feedback”: establishing mechanisms for younger employees to provide structured feedback on older employees' work plans (e.g., quarterly “reverse downward feedback” sessions), artificially creating moderate cognitive conflict to activate cognitive flexibility. Building a lifelong learning culture: incorporating “intergenerational learning participation” metrics into performance evaluations (e.g., frequency of participation in cross-generational knowledge sharing activities, progress in learning new skills), legitimizing the behavior of learning from younger colleagues as a professionally recognized career development activity.

### Limitations and future research directions

While this study offers important insights, several limitations should be acknowledged. First, we focused on cognitive flexibility as the mediating mechanism; future research could explore other pathways, particularly emotional mechanisms. In the context of knowledge counterflow, older employees may experience complex affective responses—such as status threat, face-loss concerns, or positive emotions from being valued—that could interact with cognitive processes to shape creativity.

Second, our operationalization of knowledge stickiness centered on the recipient's perceived difficulty in understanding transferred knowledge. However, stickiness is a multidimensional construct that also arises from knowledge characteristics (e.g., tacitness, complexity; Simonin, 1999), sender capabilities (Szulanski et al., 2004), intergenerational communication barriers (Schmidt and Muehlfeld, 2017), and organizational support systems (Riusala and Smale, 2007). Future research adopting multidimensional measures could illuminate whether different sources of stickiness exert distinct moderating effects—for instance, complexity-driven stickiness may be alleviated through knowledge visualization, whereas institution-driven stickiness may require incentive redesign. Additionally, other boundary conditions, such as younger employees' knowledge transfer willingness and ability, merit further investigation (Fasbender and Gerpott, 2022).

Third, our creativity measure—supervisor ratings of overall performance—did not distinguish between incremental and radical creativity (Gilson and Madjar, 2011; Madjar et al., 2011). Given that supervisor evaluations tend to capture observable, incremental behaviors, our findings likely reflect knowledge counterflow's facilitative effect on incremental creativity. Theoretically, these two creativity forms differ in cognitive demands: incremental creativity relies more on deep recombination of domain-specific knowledge (Katila and Ahuja, 2002), whereas radical creativity requires cross-domain integration and greater cognitive reframing (Taylor and Greve, 2006). We encourage future research to adopt specialized scales (e.g., Madjar et al., 2011) to test whether knowledge counterflow exerts differential effects on these two creativity types.

Fourth, our model focused on knowledge counterflow's effect on older employees and may not generalize to the reverse direction (older-to-younger transfer). Younger employees typically lack highly entrenched cognitive structures (Dane, 2010); their creative bottleneck may lie more in insufficient domain knowledge than in cognitive flexibility. Thus, the same mediational pathway may not apply to younger employees—a possibility warranting empirical examination.

## Conclusion

Cognitive entrenchment can impose significant barriers on older employees attempting to activate their creativity. Workplace managers often have negative stereotypes of these employees and provide limited resources for their development. Thus, older employees find it difficult to enhance their creativity on their own and require organizational support. We found that knowledge counterflow within an organization can form a learning network space that includes diverse knowledge and close relationships, which can effectively stimulate older employee creativity. We constructed a moderated mediation model to extend the research into the type of organizational support (knowledge counterflow) that may affect older employee creativity (through cognitive flexibility), and identified the boundary conditions under which this process influences their creativity (knowledge stickiness).Thus, we explain the conditions that can facilitate older employee creativity using a precise model. Managers can draw on our findings to establish how knowledge management can help facilitate older employee creativity.

## Data Availability

The raw data supporting the conclusions of this article will be made available by the authors, without undue reservation.
